# Do biobanks need pharmacists? Support of pharmacy students to biobanking of human biological material for pharmaceutical research and development

**DOI:** 10.3389/fphar.2024.1406866

**Published:** 2024-05-10

**Authors:** Jan Domaradzki, Anita Majchrowska, Judyta Cielecka-Piontek, Dariusz Walkowiak

**Affiliations:** ^1^ Department of Social Sciences and Humanities, Poznan University of Medical Sciences, Poznań, Poland; ^2^ Chair and Department of Humanities and Social Medicine, Medical University of Lublin, Lublin, Poland; ^3^ Department of Pharmacognosy and Biomaterials, Poznan University of Medical Sciences, Poznań, Poland; ^4^ Department of Organization and Management in Healthcare, Poznan University of Medical Sciences, Poznań, Poland

**Keywords:** biobanking, biomedical research, biospecimens, pharmacy students, research biobanks, tissue donation

## Abstract

**Objectives:**

This study aimed to assess the biobank awareness among Polish pharmacy students and how it affects their support for biobank research.

**Methods:**

A survey among 366 pharmacy students enrolled at two Polish medical universities: the Poznań University of Medical Sciences and Medical University of Lublin was conducted.

**Results:**

Although most pharmacy students felt positivity about biobanking and expressed the willingness to donate their biospecimens for biomedical research, their awareness on research biobanks was low. Their willingness to participate was driven by the desire to benefit society, help advance science and develop new therapies. While students supported donation for most types of research, biobanks run by medical universities were the highest trusted research institutions. The primary factors associated with student’s willingness to participate were religiosity and place of study. Notably, nonreligious students and those studying in Poznan exhibited more favourable attitudes toward donating for research and expressed greater support for the establishment of research biobanks in Poland.

**Conclusion:**

Since biobank awareness among future pharmacists is inadequate incorporating biobank competency domains into education and training of pharmacists is required.

## Graphical Abstract

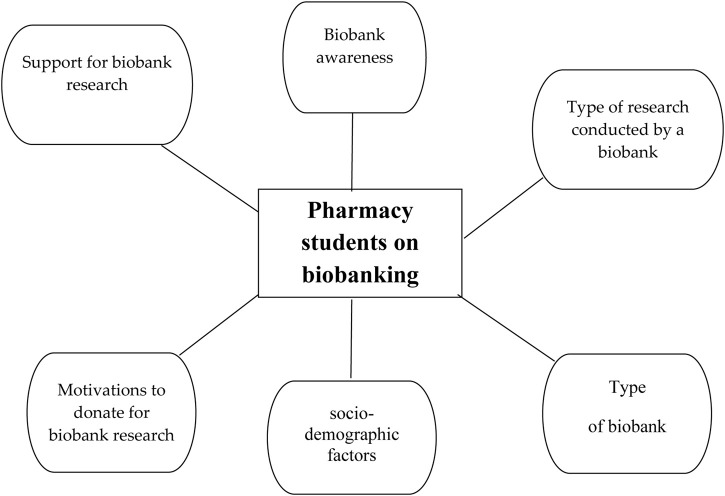

## Introduction

During past few decades there has been a huge progress in the discovery of genetic basis of many diseases and the development of new diagnostic and screening tests, as well as new drug therapies which become widely available to the public. While such progress has allowed the rapid development of personalized and precision medicine (Olson et al., 2014; Annaratone et al., 2021) it was closely related to recent advances in pharmacogenetics and pharmacogenomics, that have been revolutionized after the human genome, among other species, has been sequenced and publicized, allowing identification of numerous genetic biomarkers associated with either positive or adverse drug responses (Sadee, 2017; Van Driest and Cascorbi, 2021). In large part this progress is due to the development of research biobanks, i.e., collections of human biospecimens and associated health, medical, nutrition, lifestyle and environmental information, which enable researchers wide access to well-annotated patient samples (McCarty and Wilke, 2010; Olson et al., 2014).

At the same time, even though with more than 500 biobanks Europe leads the world in biobanking (ODonoghue et al., 2022), especially since the European biobanking research infrastructure for biobanking: the Biobanking and Biomolecular Resources Research Infrastructure (BBMRI)—European Research Infrastructure Consortium (ERIC) was launched in 2013 as a part of EU science policy and scientific collaboration (Litton, 2018; Argudo-Portal and Domènech, 2020), such progress is highly dependent on commercial involvement. In fact, although the largest collections of (human) biospecimens are typically collected by public institutions, i.e., academic medical centres or hospitals, it is usually commercial companies, including the pharmaceutical industry, that are crucial for the development of novel tests, drugs, treatments or vaccines that benefit the entire population (Biobanks need pharma, 2009; Swifka et al., 2013; Simeon-Dubach et al., 2020). Nevertheless, even though it is estimated that private sector accounts for 79%–90% of research and development of new pharmaceutical products (Chakravarthy et al., 2016), academic researchers have usually better access to biosamples stored in biobanks. Moreover, pharmaceutical industry is also challenged by lower level of social trust among the public, which can, in turn, affect people’s willingness to donate their biospecimens for research purposes, social perception of risks related to the privacy and confidentiality of biosamples, and the preference for broad consent (Master et al., 2013; Heredia et al., 2017; Sanderson et al., 2017; Domaradzki and Pawlikowski, 2019). In fact, previous research demonstrated that biobank’s preference for broad or blanket consent is not always shared by the donors who frequently opt for one-time or study-specific consent and wish to be re-contacted before their samples and annotated data could be used in future research (Gefenas et al., 2012; Caenazzo et al., 2013; D’Abramo et al., 2015). For that reason, it is suggested that based on various ethical background assumptions the donors should be offered alternative models of consent (Wiertz and Boldt, 2022), and that biobanks should stay in touch with the donors through different form of consent, including electronic informed consent (De Sutter et al., 2022). It is also stressed that the donors’ consent to biobanking alone is not enough and ethics committee approval are also require to make sure that it follows the General Data Protection Regulation, which obliges all institutions, including biobanks, to protect all personal data.

Moreover, numerous research show that while scientific or academic institutions are trusted more than commercial ones, the donors are often concerned over the possibility of selling their biosamples to pharmaceutical companies or that their samples and associated health data could be used for commercial gain. Moreover, they are also anxious about the issues related to the ownership and profit sharing (Trinidad et al., 2012; Critchley et al., 2015; Tozzo and Caenazzo, 2020). For instance, Dive et al. demonstrated that as the Australian respondents were strongly concerned about profit-motivated research, they were significantly less willing to donate to a biobank associated with a pharmaceutical company (57%) or a biotechnology firm (59%), then the one funded by the government (86%) or research institution (94%) (Dive et al., 2020). Another study found that while most Americans trusted academic and government researchers (92% and 80% respectively) fewer trusted researchers related to pharmaceutical company (75%) (Kaufman et al., 2009). Comparable results were also found in Canada, Scotland or China where most people declare trust towards hospital research and government institutions, while were few trust for-profit organizations and research companies (Treweek et al., 2009; Caulfield et al., 2012; Ma et al., 2012).

What is equally important, is that concerns over commercialization and the risk of selling one’s samples to a pharmaceutical company is also expressed by researchers (Moodley and Singh, 2016). For example, Caixeiro et al. showed that most health professionals were more likely to participate in biobank affiliated with hospital (83%), university (75%) and government (55%) research institutions than for-profit companies (20%) (Caixeiro et al., 2016).

This is of key importance since both medical professionals and researcher play a key role in disseminating social awareness about biobanks and can help recruit new donors who will share their biospecimens for research purposes (Persaud and Bonham, 2018; Chróścicka et al., 2022). For that reason, it is important to rise knowledge about biobanks not only among the general public but also healthcare professionals and medical students. Thus, this study seeks to explore the perception of Polish pharmacy students (PS) of research biobanks, including: 1) their biobank awareness, 2) willingness and motivations to share one’s biospecimens for research purposes, 3) support for various type of biobank research, 4) trust towards biobank institutions, and 5) factors associated with future pharmacists’ support for biobank research.

## Materials and methods

### Study design

Although there are several studies on the attitudes of Polish citizens towards biobanking of human biological material (HBM) for research purposes (Majchrowska et al., 2022; Pawlikowski et al., 2022; Domaradzki et al., 2023; Pronicki et al., 2023), there remains a shortage of studies on the attitudes of (future) pharmacists on research biobanks. Thus, this study was designed to assess knowledge and attitudes of Polish pharmacy students on research biobanks.

After a thorough analysis of literature an anonymous self-administered web questionnaire was developed to assess PS’ knowledge and attitudes towards participation in biobank research involving HBM.

### Ethical issues

This project was granted approval by the Poznan University of Medical Sciences Bioethics Committee (KB—926/21). The survey was performed in accordance with the ethical standards as laid down in the 1964 Declaration of Helsinki (revised in 2000) (Sawicka-Gutaj et al., 2022). All eligible students provided their informed consent.

### Participants and setting

The survey was conducted between 30th November 2022 and 30th June 2023 among PS at two Polish medical universities: the Poznan University of Medical Sciences (PUMS) and Medical University of Lublin (MUL). The rational for choosing these two universities was that while they are among the biggest medical universities in the country, pharmacy studies organized by these universities are ranked among the best in Poland (six and fourth place respectively) (Perspektywy, 2021). Additionally, Polish society is split in public attitudes towards ideological issues, religion and politics, where the Eastern part of the country (Lublin) is more conservative and feels strongly committed to religion and traditional values, and in the West (Poznan) people are more liberal and fewer believe that religion is very important. Such differences can, in turn, affect people’s attitudes towards science and biotechnology (Critchley et al., 2019; Broekstra et al., 2021).

Respondents were eligible to participate if they were aged over 18, were enrolled at medial university, were pharmacy students, were willing to participate in the survey, were able to use electronic devices and complete the online questionnaire and provided the informed consent. An invitation to participate in the study was posted on an online platform. Overall, 366 students responded and completed the survey.

### Research tool

The questionnaire used in this project was developed according to the recommendations of the European Statistical System (Eurostat Brancato et al., 2005). After conducting a literature search and wide reading on the topic a preliminary questionnaire was drafted. Than earlier instruments that were available in the literature were also reviewed and the final version of questions were phrased and designed. Next, the questionnaire was evaluated by a panel of experts (a biotechnologist, sociologist and public health specialist) and revised based on their comments. After reformulating four questions, a standardized questionnaire was developed and pre-tested via an online platform with five students. This in turn, led to rephrasing three questions. Finally, two additional specialists again evaluated the questionnaire: a biotechnologist and sociologist. Additionally, it was approved by the University Student Council Board.

The questionnaire consisted of seventeen close-ended questions designed to explore PS’ knowledge and attitudes on biobanking of HBM for research purposes. It was divided into four sections. The first explored questions regarding students’ knowledge and attitudes towards research biobanks, and asked if they were aware of such institutions, what was their apprehension of research biobanks, whether they would support establishment of a biobank in Poland and donate their biospecimens for research purposes. The second section included questions regarding type of research and type of biobank they would be willing to donate to. The third asked questions on students’ motivations and expectations related to sharing their HBM. The last section contained a series of demographic questions (Supplementary Material).

Because literature revied conducted demonstrated that the awareness on biobanking in general population is low the questions did not include technical jargon and were formulated in simple language instead. While questions used a 5-Likert scale (1 was strongly disagreement and five strongly agreement), the scores also contained ambiguous answers (“I do not know”).

### Data collection

After the permission to distribute a questionnaire was obtained from the board of both universities an invitation letter was sent to students’ group leaders, who were asked to provide their fellow students with a link to the questionnaire which was made available online via a communication platform. It contained information on the purpose of the survey and methods used, voluntary, anonymous and confidential character of the survey, and the possibility to withdraw from the study at any time without any implications. Before completing the survey all students who volunteered signed the online consent form that was placed at the beginning of the questionnaire.

The survey was collected using a self-administered web questionnaire with the assistance of their mobile devices (e.g., smartphones or tablets). It took 15–20 min to complete the survey.

### Data analysis

The data acquired from the administered questionnaires underwent meticulous scrutiny to ensure meticulousness, uniformity, and inclusiveness. Following validation, the data was encoded and subsequently imported into JASP (Version 0.17.2.1) for the execution of rigorous statistical analyses. The outcomes are depicted through descriptive statistical analyses, while the interrelationships among variables are gauged through the computation of odds ratios (OR). To ascertain the precision of OR, 95% confidence intervals (CI) were calculated. The verification of comparability in the distribution of Likert scale responses across various groups was conducted using Chi-squared tests. A significance threshold of *p* < 0.05 was adopted to establish the presence of statistical significance.

## Results

366 PS responded and completed the questionnaire, including 186 from PUMS (50.8%) and 180 from MUL (49.2%) (Table 1). The sample was dominated by female students (80.6%), while male students constituted 19.4% of respondents. However, such overrepresentation of females reflects the gender differences in the proportion across medical students in Poland (Eurostat Statistics Explained, 2022). Most students were enrolled in their first, second or third year of study (79.25%) and lived in town with less than 100,000 inhabitants (67.2%).

**TABLE 1:** Sample characteristics.

	n(%)
Gender	
women	295 (80.6)
men	71 (19.4)
Year of the study	
1–3	290 (79.2)
4–5	76 (20.8)
Domicile	
town up to 100,000 inhabitants	246 (67.2)
city with more than 100,000 inhabitants	120 (32.8)
What role does religion play in your life?	
significant and rather significant	144 (39.3)
insignificant and none	222 (60.7)
Studying at the Medical University	
Poznan	186 (50.8)
Lublin	180 (49.2)

Table 2 presents the awareness of research biobanks among PS, exploring various demographic factors and attitudes. The gender comparison revealed no significant differences, and there were no variations in responses based on the size of the town or city in which surveyed students reside. In terms of the year of study comparison, students in their 4th and 5th years were 2.38 times more aware of research biobanks compared to those in their 1st to 3rd years (95% CI: 1.288–4.390). Regarding religious affiliation, nonreligious students were 1.5 times more likely to have a positive impression related to the term “biobank”. Additionally, nonreligious students were 1.48 times more likely to express positivity about donating samples of their biological material to a biobank for research purposes (95% CI: 1.233–1.771) compared to religious students. Furthermore, nonreligious students were more inclined to support the establishment of biobanks in Poland. Interestingly, students studying in Poznań provided more affirmative responses to all four questions compared to students studying in Lublin.

**TABLE 2:** Research biobanks awareness among pharmacy students.

		Women vs. men	4–5 years students vs. 1–3 years students	Nonreligious vs. religious students	Living in cities with more than 100,000 inhabitants vs. living in towns with less than 100,000 inhabitants	Students from Poznan vs. students from Lublin
Students who have previously heard about biobanks vs. all others	OR		2.38			1.18
	95%CI		1.288–4.390			1.018–1.366
	*p*	ns	*p* = 0.004	ns	ns	*p* = 0.01
Students who have positive impression when they hear a word biobank vs. all others	OR		1.33	1.5		1.69
	95%CI		1.033–1.702	1.150–1.957		1.313–2.163
	*p*	ns	*p* = 0.01	*p* = 0.001	ns	*p* < 0.001
Students positive about donating the sample of their biological material to a biobank for research purposes vs. all others	OR			1.48		1.32
	95%CI			1.233–1.771		1.125–1.540
	*p*	ns	ns	*p* < 0.001	ns	*p* < 0.001
Should a research biobank collecting biological samples for research purposes be established in Poland?	OR			1.17		1.16
	95%CI			1.051–1.294		1.052–1.269
	*p*	ns	ns	*p* = 0.002	ns	*p* = 0.001

ns, not significant.

Table 3 offers a comprehensive and nuanced exploration of PS’ attitudes and preferences across a spectrum of biobank research domains. What lends Table 3 its particular insightfulness is the careful categorization of responses based on diverse demographic characteristics. Within the table, numerical values in each cell depict the frequency of students aligning with specific response categories for each research theme and demographic segment. The assessment of PS’ endorsement for several types of biobank research highlights religiosity as the most distinguishing factor. However, notable distinctions in responses also emerged between women and men for many of the questions. Furthermore, variations in answers were observed based on the students’ location of study. Women showed slightly lower support for research on the pathogenesis of cancer compared to men, with a statistically significant difference (χ2 = 10.311, *p* < 0.05). Additionally, 4–5 years students exhibited slightly higher support than 1–3 years students, with a statistically significant difference (χ2 = 30.191, *p* < 0.001). Research on psychiatric disorders received more support from students in Poznań than from those in Lublin (χ2 = 24.673, *p* < 0.001). Moreover, religious students displayed lower support for commercial research compared to non-religious students.

**TABLE 3:** Pharmacy students’ support for various type of biobank research.

	Women	Men	1–3 years students	4–5 years students	Nonreligious students	Religious students	Living in cities with more than 100,000 inhabitants	Living in towns with less than 100,000 inhabitants	Students from Poznan	Students from Lublin
To what type of research you would be willing to donate to?										
Research on the pathogenesis of cancer										
definitely yes	192(65.1)	57(80.3)	188(64.8)	61(80.3)	170(76.6)	79(54.9)	89(74.2)	160(65.1)	138(74.2)	111(61.7)
rather yes	83(28.1)	11(15.5)	81(27.9)	13(17.1)	48(21.6)	46(31.9)	25(20.8)	69(28)	41(22)	53(29.4)
I do not know	14(4.8)	0(0)	14(4.8)	0(0)	2(0.9)	12(8.3)	3(2.5)	11(4.5)	4(2.2)	10(5.6)
rather no	3(1)	1(1.4)	4(1.4)	0(0)	2(0.9)	2(1.4)	1(0.8)	3(1.2)	2(1.1)	2(1.1)
definitely no	3(1)	2(2.8)	3(1.1)	2(2.6)	0(0)	5(3.5)	2(1.7)	3(1.2)	1(0.5)	4(2.2)
Chi-square test	χ^2^ = 10.311, **p < 0.05**		χ^2^ = 10.698, **p < 0.05**		χ^2^ = 30.191, **p < 0.001**		χ^2^ = 3.670, ns		χ^2^ = 8.735, ns	
Research on curable somatic disease										
definitely yes	140(47.5)	44(62)	134(46.2)	50(65.8)	131(59)	53(36.8)	68(56.6)	116(47.2)	106(57)	78(43.3)
rather yes	119(40.3)	20(28.2)	116(40)	23(30.3)	79(35.6)	60(41.7)	41(34.2)	98(39.8)	67(36)	72(40)
I do not know	17(5.8)	2(2.8)	19(6.5)	0(0)	5(2.2)	14(9.7)	6(5)	13(5.3)	6(3.2)	13(7.2)
rather no	16(5.4)	0(0)	15(5.2)	1(1.3)	7(3.2)	9(6.3)	3(2.5)	13(5.3)	6(3.2)	13(7.2)
definitely no	3(1)	5(7)	6(2.1)	2(2.6)	0(0)	8(5.5)	2(1.7)	6(2.4)	1(0.5)	7(3.9)
Chi-square test	χ^2^ = 18.942, **p < 0.001**		χ^2^ = 13.212, **p = 0.01**		χ^2^ = 33.054, **p < 0.001**		χ^2^ = 3.798, ns		χ^2^ = 12.425, **p < 0.05**	
Research on psychiatric disorders, i.e., schizophrenia, depression										
definitely yes	163(55.3)	46(64.8)	156(53.8)	53(69.7)	151(68)	58(40.3)	81(67.5)	128(52)	125(67.2)	84(46.7)
rather yes	88(29.8)	15(21.1)	91(31.4)	12(15.8)	56(25.2)	47(32.6)	25(20.8)	78(31.7)	37(19.9)	66(36.7)
I do not know	23(7.8)	6(8.5)	26(9)	3(3.9)	6(2.7)	23(16)	7(5.8)	22(8.9)	12(6.5)	17(9.4)
rather no	15(5.1)	1(1.4)	10(3.4)	6(7.9)	9(4.1)	7(4.9)	5(4.2)	11(4.5)	11(5.9)	5(2.8)
definitely no	6(2)	3(4.2)	7(2.4)	2(2.6)	0(0)	9(6.2)	2(1.7)	7(2.8)	1(0.5)	8(4.4)
Chi-square test	χ^2^ = 5.369, ns		χ^2^ = 12.530, **p < 0.05**		χ^2^ = 46.891, **p < 0.001**		χ^2^ = 8.225, ns		χ^2^ = 24.673, **p < 0.001**	
Research on intelligence										
definitely yes	110(37.3)	38(53.5)	115(39.7)	33(43.4)	112(50.5)	36(25)	47(39.2)	101(41)	82(44.1)	66(36.7)
rather yes	103(34.9)	15(21.1)	95(32.8)	23(30.3)	72(32.4)	46(31.9)	34(28.3)	84(34.1)	58(31.2)	60(33.3)
I do not know	32(10.8)	5(7)	30(10.3)	7(9.2)	15(6.8)	22(15.3)	14(11.7)	23(9.4)	19(10.2)	18(10)
rather no	40(13.6)	7(9.9)	39(13.4)	8(10.5)	21(9.5)	26(18.1)	21(17.5)	26(10.6)	25(13.4)	22(12.2)
definitely no	10(3.4)	6(8.5)	11(3.8)	5(6.6)	2(0.9)	14(9.7)	4(3.3)	12(4.9)	2(1.1)	14(7.8)
Chi-square test	χ^2^ = 11.887, **p < 0.05**		χ^2^ = 1.874, ns		χ^2^ = 40.844, **p < 0.001**		χ^2^ = 4.802, ns		χ^2^ = 10.887, **p < 0.05**	
Research on aggression and violence										
definitely yes	110(37.3)	37(52.1)	112(38.6)	35(46)	109(49.1)	38(26.4)	50(41.6)	97(39.4)	85(45.7)	62(34.4)
rather yes	119(40.3)	18(25.4)	110(37.9)	27(35.5)	84(37.8)	53(36.8)	45(37.5)	92(37.4)	67(36)	70(38.9)
I do not know	28(9.5)	10(14.1)	33(11.4)	5(6.6)	12(5.4)	26(18.1)	12(10)	26(10.6)	17(9.1)	21(11.7)
rather no	29(9.8)	2(2.8)	26(9)	5(6.6)	15(6.8)	16(11.1)	11(9.2)	20(8.1)	15(8.1)	16(8.9)
definitely no	9(3.1)	4(5.6)	9(3.1)	4(5.3)	2(0.9)	11(7.6)	2(1.7)	11(4.5)	2(1.1)	11(6.1)
Chi-square test	χ^2^ = 12.126, **p < 0.05**		χ^2^ = 3.453, ns		χ^2^ = 37.823, **p < 0.001**		χ^2^ = 2.015, ns		χ^2^ = 10.253, **p < 0.05**	
Research on reproductive cloning										
definitely yes	37(12.5)	25(35.2)	47(16.2)	15(19.7)	48(21.6)	14(9.7)	23(19.2)	39(15.9)	40(21.5)	22(12.2)
rather yes	39(13.2)	14(19.7)	44(15.2)	9(11.8)	37(16.7)	16(11.1)	15(12.5)	38(15.4)	25(13.5)	28(15.5)
I do not know	40(13.6)	6(8.5)	35(12.1)	11(14.5)	25(11.3)	21(14.6)	17(14.1)	29(11.8)	21(11.3)	25(13.9)
rather no	79(26.8)	8(11.3)	76(26.2)	11(14.5)	54(24.3)	33(22.9)	24(20)	63(25.6)	41(22)	46(25.6)
definitely no	100(33.9)	18(25.3)	88(30.3)	30(39.5)	58(26.1)	60(41.7)	41(34.2)	77(31.3)	59(31.7)	59(32.8)
Chi-square test	χ^2^ = 27.306, **p < 0.001**		χ^2^ = 6.225, ns		χ^2^ = 16.545, **p < 0.01**		χ^2^ = 2.643, ns		χ^2^ = 5.934, ns	
Research into incurable genetic diseases										
definitely yes	175(59.3)	53(74.6)	170(58.6)	58(76.3)	159(71.6)	69(47.9)	87(72.5)	141(57.3)	128(68.8)	100(55.6)
rather yes	100(33.9)	11(15.5)	97(33.5)	14(18.4)	58(26.1)	53(36.8)	28(23.3)	83(33.8)	48(25.8)	63(35)
I do not know	11(3.7)	4(5.6)	14(4.8)	1(1.3)	1(0.5)	14(9.7)	2(1.7)	13(5.3)	6(3.2)	9(5)
rather no	6(2)	2(2.8)	7(2.4)	1(1.3)	4(1.8)	4(2.8)	2(1.7)	6(2.4)	4(2.2)	4(2.2)
definitely no	3(1)	1(1.4)	2(0.7)	2(2.7)	0(0)	4(2.8)	1(0.8)	3(1.2)	0(0)	4(2.2)
Chi-square test	χ^2^ = 9.297, ns		χ^2^ = 11.733, **p < 0.05**		χ^2^ = 36.032, **p < 0.001**		χ^2^ = 8.771, ns		χ^2^ = 9.970, **p < 0.05**	
Commercial research										
definitely yes	23(7.8)	11(15.5)	29(10)	5(6.6)	27(12.2)	7(4.9)	13(10.8)	21(8.5)	19(10.2)	15(8.3)
rather yes	34(11.5)	6(8.4)	35(12.1)	5(6.6)	27(12.2)	13(9)	8(6.7)	32(13)	21(11.3)	19(10.6)
I do not know	55(18.6)	10(14.1)	54(18.6)	11(14.5)	35(17.7)	30(20.8)	18(15)	47(19.1)	30(16.1)	35(19.4)
rather no	99(33.6)	20(28.2)	91(31.4)	28(36.8)	78(35.1)	41(28.5)	43(35.8)	76(30.9)	62(33.3)	57(31.7)
definitely no	84(28.5)	24(33.8)	81(27.9)	27(35.5)	55(24.8)	53(36.8)	38(31.7)	70(28.5)	54(29.1)	54(30)
Chi-square test	χ^2^ = 5,876, ns		χ^2^ = 4.733, ns		χ^2^ = 12.537, **p < 0.05**		χ^2^ = 5.078, ns		χ^2^ = 1.067, ns	

Statistically significant differences are written in boldface; ns, not significant.

Table 4 explores PS’ motivations for donating their biological samples for research purposes. These motivations encompass the desire to benefit society and future generations, contribute to scientific progress and therapy development, support family and relatives, receive medical treatment or services, understand their own health status, and receive financial compensation. It also shows that although most participants were drive by altruistic motives, still many perceived donations as a kind of the reciprocity game and expected recognition, access to research findings, disclosure of personal health information or financial remuneration. Students also differed in their opinions on whether biobank donors should receive financial compensation and who should own the rights to the donated samples.

**TABLE 4:** Pharmacy students’ motivations for participation in research biobank.

	Women	Men	1–3 years students	4–5 years students	Nonreligious students	Religious students	Living in cities with more than 100,000 inhabitants	Living in towns with less than 100,000 inhabitants	Students from Poznan	Students from Lublin
What would be your primary motivation for donating your biological material to a research biobank?										
to benefit society and future generations	19(6.7)	4(5.9)	18(6.5)	5(6.6)	11(5)	12(9)	7(6.1)	16(6.8)	11(6.1)	12(7.1)
progress in science, helping in generating new knowledge and development of therapies for various diseases	185(65.1)	52(76.5)	182(65.9)	55(72.4)	158(72.1)	79(59.4)	83(72.2)	154(65)	132(72.5)	105(61.8)
to benefit my family, relatives and mine own	34(12)	5(7.3)	32(11.6)	7(9.2)	16(7.3)	23(17.3)	8(6.9)	31(13.1)	15(8.2)	24(14.1)
the desire to receive medical treatment/service	1(0.4)	1(1.5)	2(0.7)	0(0)	0(0)	2(1.5)	0(0)	2(0.8)	0(0)	2(1.2)
the desire to know my health status	39(13.7)	6(8.8)	37(13.4)	8(10.5)	29(13.2)	16(12)	16(13.9)	29(12.2)	22(12.1)	23(13.5)
the desire to receive financial gratification	6(2.1)	0(0)	5(1.8)	1(1.3)	5(2.3)	1(0.8)	1(0.9)	5(2.1)	2(1.1)	4(2.3)
Chi-square test	χ^2^ = 5.836, ns		χ^2^ = 1.695, ns		χ^2^ = 15.999, **p < 0.01**		χ^2^ = 5.108, ns		χ^2^ = 7.485, ns	
What would you expect in exchange of donation samples of your biological material to a research biobank?										
acknowledgment	2(0.7)	2(2.8)	4(1.4)	0(0)	4(1.8)	0(0)	4(3.3)	0(0)	3(1.6)	1(0.6)
research results	29(9.8)	15(21.2)	33(11.4)	11(14.5)	32(14.4)	12(8.3)	19(15.9)	25(10.1)	24(12.9)	20(11.1)
personal health information	233(79)	46(64.8)	220(75.9)	59(77.7)	172(77.5)	107(74.3)	81(67.5)	198(80.5)	147(79)	132(73.3)
financial gratification	19(6.4)	4(5.6)	20(6.9)	3(3.9)	9(4.1)	14(9.7)	12(10)	11(4.5)	7(3.8)	16(8.9)
nothing	12(4.1)	4(5.6)	13(4.5)	3(3.9)	5(2.3)	11(7.6)	4(3.3)	12(4.9)	5(2.7)	11(6.1)
Chi-square test	χ^2^ = 10.363, **p < 0.05**		χ^2^ = 2.426, ns		χ^2^ = 15.660, **p < 0.01**		χ^2^ = 16.505, **p < 0.01**		χ^2^ = 7.846, ns	
Do you think donors should receive financial compensation for donating samples?										
yes	103(34.9)	20(28.2)	93(32.1)	30(39.5)	75(33.9)	48(33.3)	44(36.7)	79(32.1)	53(28.5)	70(38.9)
no	62(21)	21(29.6)	71(24.5)	12(15.8)	43(19.3)	40(27.8)	21(17.5)	62(25.2)	44(23.7)	39(21.7)
I do not know	130(44.1)	30(42.2)	126(43.4)	34(44.7)	104(46.8)	56(38.9)	55(45.8)	105(42.7)	89(47.8)	71(39.4)
Chi-square test	χ^2^ = 2.667, ns		χ^2^ = 3.012, ns		χ^2^ = 3.994, ns		χ^2^ = 2.791, ns		χ^2^ = 4.579, ns	
Who should profit from the biobank research?										
sponsor of the research/biobank owner	64(21.7)	19(16.8)	57(19.6)	26(34.2)	58(26.1)	25(17.4)	31(25.8)	52(21.1)	57(30.6)	26(14.4)
donors	8(2.7)	6(8.4)	13(14.5)	1(1.3)	4(1.8)	10(6.9)	3(2.5)	11(4.5)	5(2.7)	9(5)
both biobank and donor	164(55.6)	25(35.2)	156(53.8)	33(43.4)	111(50)	78(54.2)	62(51.7)	127(51.6)	85(45.7)	104(57.8)
I do not know	59(20)	21(29.6)	64(22.1)	16(21.1)	49(22.1)	31(21.5)	24(20)	56(22.8)	39(21)	41(22.8)
Chi-square test	χ^2^ = 12.580, **p < 0.01**		χ^2^ = 8.488, **p < 0.05**		χ^2^ = 9.303, **p < 0.05**		χ^2^ = 1.886, ns		χ^2^ = 14.587, **p < 0.01**	
Who should own the rights to the samples donated to the biobank										
biobank	31(10.5)	7(9.9)	25(8.6)	13(17.1)	25(11.3)	13(9)	15(12.5)	23(9.4)	24(12.9)	14(7.8)
donors	57(19.3)	23(32.4)	67(23.1)	13(17.1)	50(22.5)	30(20.8)	31(25.8)	49(19.9)	36(19.3)	44(24.4)
both biobank and donor	191(64.7)	38(53.5)	181(62.4)	48(63.2)	136(61.3)	93(64.6)	72(60)	157(63.8)	119(64)	110(61.1)
I do not know	16(5.4)	3(4.2)	17(5.9)	2(2.6)	11(4.9)	8(5.6)	2(1.7)	17(6.9)	7(3.8)	12(6.7)
Chi-square test	χ^2^ = 5.808, ns		χ^2^ = 6.382, ns		χ^2^ = 0.748, ns		χ^2^ = 6.523, ns		χ^2^ = 5.004, ns	

Statistically significant differences are written in boldface; ns: not significant.

The most distinguishing factor in respondents’ answers to the questions within this table was their religious attitude. While 72.1% of non-religious students declared that their primary motivation for participation in biobank research would be the desire to help advance science and the development of therapies for various diseases, among religious students it was 59.4%. On the other hand, 7.3% of non-religious students mentioned benefiting their family, relatives and themselves as their primary motivation, while for religious students it was 17.3%. Moreover, responses to the question regarding beneficiaries of biobank research profits diverged among diverse groups based on factors such as gender, academic year, and the city where the respondents studied. Significant variations were also noted among students from various academic years concerning their motivation to contribute to societal and future generational benefits (χ2 = 15.999, *p* < 0.01). Additionally, notable differences were observed among students from different cities regarding their expectations to receive research results (χ2 = 16.505, *p* < 0.01). Furthermore, significant disparities were detected in the responses of students from diverse cities regarding who should benefit from biobank research (χ2 = 14.587, *p* < 0.01).

Table 5 provides a thorough examination of PS’ levels of trust in various biobank institutions. Gender comparison revealed no significant differences. However, when comparing the year of study, senior students (4–5 years) showed a moderate inclination to trust medical university-led research biobanks (OR: 1.097, 95% CI: 1.016–1.184) and exhibited heightened trust in foreign biobanks (OR: 1.272, 95% CI: 1.066–1.517). Religiosity also influenced trust levels, with nonreligious students demonstrating greater trust in donating to research biobanks across various categories, including those run by medical university (OR: 1.177, 95% CI: 1.072–1.292) and foreign biobanks (OR: 1.412, 95% CI: 1.160–1.718). Urban residency played a role as well, as students living in larger cities (more than 100,000 inhabitants) expressed a higher level of trust in biobanks run by medical university (OR: 1.084, 95% CI: 1.005–1.170). Moreover, differences among students from different cities were revealed. Those from Poznan were more likely to trust medical biobanks run by medical university compared to their counterparts from Lublin. Specifically, students from Poznan were significantly more willing to donate to a biobank run by a public clinical hospital (OR = 1.148, 95% CI: 1.011–1.304, *p* < 0.05). Additionally, nonreligious students showed a significantly higher inclination to donate to a biobank led by a Polish pharmaceutical company (OR = 1.193, 95% CI: 1.017–1.40, *p* < 0.05).

**TABLE 5:** Pharmacy students trust towards biobank institutions.

		Women vs. men	4–5 years students vs. 1–3 years students	Nonreligious vs. religious students	Living in cities with more than 100,000 inhabitants vs. Living in towns with less than 100,000 inhabitants	Students from Poznan vs. students from Lublin
Would you donate the sample of your biological material to a research biobank led by						
medical university	OR		1.097	1.177	1.084	1.199
	95%CI		1.016–1.184	1.072–1.292	1.005–1.170	1.104–1.303
	*p*	ns	*p* < 0.01	*p* < 0.001	*p* < 0.05	*p* < 0.001
public clinical hospital	OR					
	95%CI					
	*p*	ns	ns	ns	ns	ns
private clinical hospital	OR			1.148		
	95%CI			1.011–1.304		
	*p*	ns	ns	*p* < 0.05	ns	ns
Polish biobank	OR					
	95%CI					
	*p*	ns	ns	ns	ns	ns
foreign biobank	OR		1.272	1.412		
	95%CI		1.066–1.517	1.160–1.718		
	*p*	ns	*p* < 0.01	*p* < 0.001	ns	ns
private biobank	OR			1.258		
	95%CI			1.019–1.552		
	*p*	ns	ns	*p* < 0.05	ns	ns
public biobank	OR					
	95%CI					
	*p*	ns	ns	ns	ns	ns
Polish pharmaceutical company	OR				1.193	
	95%CI				1.017–1.40	
	*p*	ns	ns	ns	*p* < 0.05	
foreign pharmaceutical company	OR			1.412		
	95%CI			1.169–1.706		
	*p*	ns	ns	*p* < 0.001	ns	ns

ns, not significant.

Table 6 presents an analysis of factors influencing PS’ willingness to donate biological samples to research biobanks based on various demographic and institutional considerations. Firstly, there were no significant differences observed between women and men in their willingness to donate samples to biobanks accessed only by researchers from the institution one donated to. However, when comparing students from different academic years, senior students (4–5 years) showed a slightly higher inclination (OR: 1.084, 95% CI: 1.028–1.144, *p* = 0.002) compared to junior students (1–3 years). Moreover, there was a significant difference between nonreligious and religious students regarding their willingness to donate samples accessible to researchers from Polish scientific institutions (OR: 1.096, 95% CI: 1.005–1.194, *p* < 0.05). Nonreligious students exhibited a higher propensity to donate in this scenario. Similarly, urban residency played a role, with students living in larger cities (more than 100,000 inhabitants) showing a significantly higher willingness to donate samples accessible to researchers from Polish commercial companies, including pharmaceuticals (OR: 1.236, 95% CI: 1.008–1.516, *p* < 0.05), foreign scientific institutions (OR: 1.325, 95% CI: 1.166–1.505, *p* < 0.001), and foreign commercial companies, including pharmaceutical industry (OR: 1.451, 95% CI: 1.142–1.842, *p* = 0.001). However, no significant differences were found regarding the willingness to donate samples accessible to researchers from Polish and foreign insurance companies across all demographic and institutional comparisons.

**TABLE 6:** Pharmacy students’ willingness to share their biospecimens in relation to various types of biobanks.

		Women vs. men	4–5 years students vs. 1–3 years students	Nonreligious vs. religious students	Living in cities with more than 100,000 inhabitants vs. Living in towns with less than 100,000 inhabitants	Students from Poznan vs. students from Lublin
Would you donate to a research biobank if your biological samples would be accessible to						
only researchers from the institution one donated to	OR			1.084		1.048
	95%CI			1.028–1.144		1.003–1.096
	*p*	ns	ns	*p* = 0.002	ns	*p* < 0.05
researchers from Polish scientific institutions	OR			1.096		1.097
	95%CI			1.005–1.194		1.014–1.187
	*p*	ns	ns	*p* < 0.05	ns	*p* = 0.01
researchers from Polish commercial companies, including pharmaceutical	OR				1.236	
	95%CI				1.008–1.516	
	*p*	ns	ns	ns	*p* < 0.05	ns
researchers from foreign scientific institutions	OR			1.325		
	95%CI			1.166–1.505		
	*p*	ns	ns	*p* < 0.001	ns	ns
researchers from foreign commercial companies, including pharmaceutical industry	OR			1.451		
	95%CI			1.142–1.842		
	*p*	ns	ns	*p* = 0.001	ns	ns
Polish and foreign insurance companies	OR					
	95%CI					
	*p*	ns	ns	ns	ns	ns

ns, not significant.

## Discussion

As the hitherto research indicates, social approval for participation in biobanking for scientific purposes seems to be relatively high both in Europe and worldwide (Ahram et al., 2012; Sanderson et al., 2017; Domaradzki and Pawlikowski, 2019). Nevertheless, in-depth analyses of attitudes towards biobanking show a range of impediments perceived by potential human body materials (HBM) donors. The obstacles include ethical, legal and religious issues (ELRI) (Master et al., 2013; Friedman et al., 2015; Heredia et al., 2017; Davis et al., 2019; Grežo and Sedlár, 2023). In spite of the dynamic growth of biobanks observed in recent years, still there are numerous challenges for them regarding the guarantee of security for the specimens stored and information related to them, character of consent to utilize HBM for research and establishing social trust in the activity of modern biobanks (Goisauf et al., 2019). Furthermore, as noted by Caenazzo and Tozzo, there are new issues that must be addressed in the future of biobanks: material transfer agreements, intellectual property, access to samples and data, ownership and custody of data and samples, return of results and incidental findings (Caenazzo and Tozzo, 2020). Big data, and artificial intelligence in biobanking are the newest among current challenges for biobanks (Caenazzo et al., 2015; Kinkorová and Topolčan, 2020; Kargl et al., 2022; Tozzo et al., 2023; Akyüz et al., 2024). To fulfil the mission, biobanks require deepened collaboration of different social subjects including politicians, policymakers, researchers, medical professionals, patients, and the public.

A significant role in promoting activities of biobanks should be played by medical professionals (Persaud and Bonham, 2018; Chróścicka et al., 2022), the healthcare system being a type of “spokespersons” of the path of development in medicine. Then they should also act like intermediaries between participants of the healthcare system and biobanks and their networks. Although surveys of medical professionals’ opinions and attitudes toward biobanking are not as common as surveys of public opinions of the issue, they demonstrate that healthcare professionals support the idea of biobanking, express willingness to collaborate with biobanks and are eager to donate their own biological material for research. Frequently, they do not show a satisfactory level of knowledge on biobanking (Caixeiro et al., 2016; Lhousni et al., 2019; Buhmeida et al., 2022).

A special area of biobanking activity is now being opened for pharmacists due to the development of genetic biobanks. Knowledge of pharmacogenetics, which analyzes individual responses of the body to pharmacotherapy instituted, allows monitoring potential health risks and minimizing their occurrence. Thus, it contributes to an increase in effectiveness of specific drugs by individual adjustment to patients’ needs. Owing to studying the relationship between drug metabolism and response to the therapy applied and genetic factors, prediction of the response of a person’s body to a specific drug is feasible based on the results of genetic tests. Pharmacogenetic research allows the development of personalized therapy tailored to patients’ individual needs; thus, such therapies may be more effective and have fewer adverse reactions than the standard treatment regimen used. In the process, the role of a pharmacist is indisputable (Nagy et al., 2020; Wang et al., 2020) so understanding the idea of biobanking is of great significance in the pharmaceutical community along with willingness to support it by both current and future professionals in the discipline. The dynamic development of pharmacogenetics and pharmacogenomics (McCarty and Wilke, 2010) in which technologies of an analysis of the entire genome is applied to research differences in pharmacological responses to work out new molecularly targeted drugs constitute a formidable challenge, and simultaneously an opportunity for pharmacy as a scientific discipline and the entire community of pharmacists. The research conducted among pharmacists-to-be and currently working pharmacists worldwide indicates that they report deficits of knowledge in this area (Makrygianni et al., 2023) and express their need for its acquisition and expansion (Albassam et al., 2018; Rahma et al., 2020) simultaneously they perceive a chance of development of pharmacy related to genetic research (Tuteja et al., 2013; Mehtar et al., 2022). Establishing comprehension of biobanking as early as during studies of pharmacy shapes pharmacists who are aware of their role and appreciate the significance of genetic research for the development of effective and precise pharmacotherapy.

The hitherto research into biobanking among future medical professionals leads to interesting conclusions. A high level of acceptance of participation in biobanking and donation of their own HBM do not correspond with an equally high level of knowledge on biobanking. Polish research performed among students of different medical studies revealed deficits of knowledge on biobanking (Krajewska-Kułak et al., 2011), its specificity and role in modern medicine along with relatively high eagerness of donation of their own tissue to biobanks (Domaradzki et al., 2023). Similar results were obtained in the research carried out among future health and care professionals (HCPs) in Saudi Arabia (Merdad et al., 2017) and Egypt (Ziady et al., 2017; Abdelhafiz et al., 2021). Moreover, a high level of acceptance along with a low level of knowledge were found in research among students of non-medical studies in Italy (Aleni et al., 2022) and Jordan (Khatib et al., 2021), where willingness to donate their own specimens to biobanks was considerably higher among students of medical studies than students of non-medical studies. Similar results were found in previous studies regarding Italian university students’ awareness on biobanking and DNA profiling. Despite the respondents’ unfamiliarity with the topics explored, there is a general agreement to participate in a biobanking for research purposes (Tozzo et al., 2017). Also recent study conducted among adult Poles showed that the biobank awareness among adult Polish citizens is low, as only 20.9% were familiar with the idea of biobanking. However, 65.3% declared the willingness to share their biospecimens and annotated data for biobank research (Pronicki et al., 2023).

The authors’ own research performed among students of pharmacy indicates that, similarly to the research, pharmacists-to-be express positive attitudes towards biobanking for scientific purposes and they would be willing to participate by donation of their own HBM. Students of higher years of studies and thus showing more advances in medical sciences both heard about biobanking for scientific purposes more frequently and had more positive associations with it. Therefore, a higher level of medical knowledge can be assumed to correlate positively with an awareness of biobanks occurrence and positive attitudes towards them as well as eagerness to donate their own HBM.

A differentiating factor of tendency of HBM donation is worth mentioning, namely, it is potentially a type of research conducted on the tissue donated. In students the greatest acceptance was found for research into pathogenesis of both neoplastic diseases and other diseases, namely, currently untreatable genetic diseases. However, considerably lower motivation to donation was found in research into treatable somatic diseases, mental diseases, intelligence, aggression, and violence. Therefore, the following conclusion can be drawn that progress of medicine in improvement of therapy effectiveness of some categories of diseases constitutes the most important argument for development of biobanks for future pharmacists. Moreover, statistically significant differences were found between students of first-year studies and students of late-year studies so it can be stated again that a level of professional/medical-specialist knowledge can be a significant variable differentiating attitudes. This supports validity of implementation of biobanking education in the curricula of medical studies and even demonstrates urgent necessity for systematic biobanking education introduction to medical studies curricula (Feero and Green, 2011). Such initiatives have been taken by some countries like Germany, Austria, France, and Canada by offering future HCPs a possibility of expanding knowledge on biobanking via differentiated forms of education at different stages of education (Gormally et al., 2017; Castellanos-Uribe et al., 2020; Kinkorová, 2021). Students taking part in the pilot study called the EduBRoTHER in the Czech Republic and Germany assessed positively the idea of biobanking education perceiving its application in their future professional career (Seidler et al., 2023).

In the authors’ own research, most of the students have a positive attitude towards biobanking, and sociodemographic variables solely differentiate the strength of their attitudes. However, the key variable that requires special attention is religiosity of the respondents as it determines all the components of the attitude towards biobanking. The non-religious students had more positive associations with the word *biobank,* were more willing to donate their own HBM to research that regarded by public opinion as controversial, namely, genetic studies of mental diseases, aggression, violence or cloning. Simultaneously, progress in medicine and development of effective therapies were a more crucial argument for non-religious students than for those who declared religiosity. Therefore, the essential role of religion highlighted in numerous previous studies is confirmed to be a modifying factor of the attitude towards biobanking (Ahram et al., 2014; Eisenhauer and Arslanian-Engoren, 2016) and generally towards scientific research (Rahma et al., 2020).

In the process of decision making on donation of tissues for research, individual motivation plays an important role. In the population studies, altruistic motives were identified as most essential ones and related to eagerness to help the sick (Overby et al., 2015; Dixon-Woods et al., 2017; Broekstra et al., 2022). Becoming a donor was perceived as contribution to public good and advantages for the entire community (Nobile et al., 2013; Domaradzki and Pawlikowski, 2019), constituting a type of social obligation. However, for the students of pharmacy researched the most significant motivation to their HBM donation was not related to widely understood benefits for the society and future generations but progress in science and participation in discovery of new methods of treatments of different diseases. This type of motivation was also identified in the research by Heredia et al. (Heredia et al., 2017) and Lewis et al. (Lewis et al., 2013). What seems to be interesting is the fact that apart from religiosity none of the variables differentiated students’ motivation declared.

Interestingly, while usually biobanks do not compensate donors for sharing their HBM, and it is often argued that remuneration would diminish donation understood as altruistic and prosocial act (Grežo and Sedlár, 2023), this research shows that many future pharmacists expected some form of renumeration, including financial gratification. This is line with previous studies showing that there are group of donors who believe that they should be compensated and paid for donating tissue for research purposes (Allen et al., 2018). Moreover, some authors argue that because increasingly biobanks become commercialized and create possibility of using the human body as a capital resource that can be patented, sold and bought (Domaradzki, 2019; Pawlikowska et al., 2023), the donors should be protected against the systematic and institutional exploitation of their altruistic motivations (Reichardt, 2010; Caulfield et al., 2014; Wendler, 2020). For example, Pawlikowska et al. (Pawlikowska et al., 2023) suggest that even though remuneration entails some risks, both for the donors and society, there are strong arguments for donors’ renumeration, especially if one’s biospecimens is going to be used by pharmaceutical and biotechnological industry.

Another important variable in the process of decision making on HBM donation for research is donors’ trust in biobank as an institution and its representatives and researchers. Therefore, there is indisputable necessity for building social trust in biobanks’ activities and research initiatives undertaken by them. Credibility and faith result from the transparent policy of biobanks in the scope of HBM collection, storage and use as well as quality of consent obtained from potential donors along with transparent communication with donors and other partners involved in the process (Gille et al., 2020; Samuel et al., 2022).

Based on the research conducted so far, it is clearly shown that trust in biobanks correlates positively with willingness to HBM donation (Critchley et al., 2015; Heredia et al., 2017; Sanderson et al., 2017). In the authors’ own research, the pharmacists-to be declared different levels of motivation to HBM donation dependent on the subject managing a biobank and further availability of tissues for categories of researchers. This means that trust/faith in a particular institution is of great significance in the decision-making process concerning donation. The respondents have the greatest trust in academic biobanks; though, a significant role of religiosity should be mentioned again. Since religiosity correlated with a lower level of trust in private clinical hospitals and foreign pharmaceutical companies and even those managed by medical universities. In turn, the year of studies correlated positively with a higher level of trust both for foreign and Polish biobanks run by medical universities. Basically, the results obtained are not different from those in other countries. To generalize, public institutions can be said to be trusted more than private ones, state ones are given greater trust than foreign ones while non-profit organizations are trusted more than commercial ones (Master et al., 2013; Ahram et al., 2014; Spector-Bagdady et al., 2018). It is worth mentioning that the greatest trust is placed in medical universities and associated with them teaching hospitals by potential donors. This is an essential information showing how medical universities can have a pivotal role in the process of biobanking development and genetic research related to them as well as in the creation of a desired image of the institutions. Studies are a period of socializing for future professionals so at this stage it is crucial for HCPs-to-be to obtain a satisfactory level of knowledge on biobanking. Innovative, multidisciplinary, and simultaneously well-organized knowledge encompassing both biomedical achievements and ehtical, legal and social issues (ELSI) regarding biobanking should shape awareness and comprehension of the path of personalized and precision medicine (Olson et al., 2014; Coppola et al., 2019; Annaratone et al., 2021).

Analyzing the research results obtained, another issue is worth highlighting, namely, a high level of complexity of the decision-making process of HBM donation and the essential role of more difficult cultural/worldview factors to be identified in the process. The research was conducted in two academic settings located in different parts of Poland - culturally and economically completely different ones. The statistical analysis allowed identification of crucial differences in attitudes towards biobanking in the students from Poznań and Lublin. They concerned both a level of knowledge and feelings regarding biobanking and their general attitude towards donation as well as a level of trust in biobanks—in particular those that do not belong to state institutions. The research results on social cohesion in Poland indicate the occurrence of essential social differences between the regions analyzed. They refer to, among other things, a level of social poverty, trust in people and public institutions, satisfaction with life and religiosity (Główny Urząd Statystyczny, 2018). Therefore, the following conclusion may be drawn that social capital determined by the aforementioned factors can play a pivotal role in shaping attitudes towards biobanking, and HBM donation for scientific purposes constitutes such a complicated process that it requires in-depth scientific research. Thus, the results obtained should be analyzed in a wider social and cultural context and diagnosis of social capital of the specific environment/place should designate specificity of communication between biobanks and potential donors. The authors’ own research confirmed the influence of such variables like a level of medical knowledge and religiosity towards attitudes to biobanks and biobanking, which had been proved in the previous research (Lewis et al., 2013; Kaufman et al., 2016; Abu Farha et al., 2020). The in-depth analysis depicts that the variables should not be interpreted separated from the social and cultural context and social capital of the specific environment that determines a level of trust and quality of social relationships.

### Study limitations

This study has some limitations. The response rate was relatively low and students at only two Pharmacy Faculties in Poland participated in the study. Consequently, our results cannot be generalized to the entire population of pharmacy students in the country. Moreover, due to the online request for participation and format of the study, it may have not available to all students. For both these reasons, a further a more in-depth study is required. Additionally, due to self-administered nature of this survey respondents were not able to ask for clarification of unclear questions and there is a risk that some issues were misinterpreted. Moreover, due to data collection method there was no control over who actually fills out the questionnaire. Finally, such study design may lead to another bias resulting from that fact that respondents could have read the questionnaire before filling it out. There is also age bias since most questionnaires were completed by younger students enrolled in their first, second or third year of study. Finally, there is possible implicit gender bias since most questionnaires were completed by female students. However, it should be acknowledged that this overrepresentation of female respondents reflects the gender differences in the proportion across medical students in Poland. Finally, it should be bear in mind, that since this study assessed only students’ declarations regarding their support in biobank research such hypothetical participation can differ significantly from actual decisions about donation.

## Conclusion

The research carried out among the students of pharmacy leads to interesting conclusions. It turns out that even for HCPs-to-be the process of decision making on HBM donation to biobanks is complicated and multifaceted.

Although the general level of acceptance for biobanking is relatively high, it differentiates significantly depending on the respondents’ features such as their level of religiosity, year of studies and the academic setting in which the research was conducted along with lower significance of gender and place of residence. This allows the formulation of the hypothesis that social capital conditioned by knowledge, level of trust in institutions, economic and social resources, and level of religiosity constitutes a pivotal factor affecting the attitude towards biobanking. The diagnosis of the capital should be a starting point for planning of targeted social campaigns while the establishment of trust in institutions that manage biobanks must be its indispensable element.

The fact that a level of medical knowledge differentiated the students’ attitude confirms necessity for the implementation of biobanking education to medical curricula. Moreover, the education should be covered according to the ELSI so as to shape aware, reliable and trustworthy HPCs who would be able to be spokespersons for both patients’ and biobanks’ interests.

The response to the question included in the title of the work seems to be simple both in the context of modern changes in medicine and the research results obtained. Indeed, pharmacy requires biobanks because due to genetic research of HBM collected pharmacogenetics can refine more effective and personalized therapies. Simultaneously, biobanks need pharmacy and pharmacists who can be reliable spokespersons promoting development of genetic research into drugs, building trust in patients and their understanding of the path in the development of medicine. As the research proves a recruiter plays a pivotal role in the decision-making process concerning participation in biobanking and HBM donation (Bosisio et al., 2021). Biobanks require specialists who while working in their environment or place of residence (e.g., community pharmacists) could build bridges of trust between patients, HBM donors and biobanks that need the material to perform systematic scientific research.

## Data Availability

The raw data supporting the conclusion of this article will be made available by the authors, without undue reservation.

## Abbreviations

BBMRI-ERIC, Biobanking and BioMolecular resources Research Infrastructure-European Research Infrastructure Consortium; HBM, human biological material; PUMS, Poznan University of Medical Sciences; MUL, Medical University of Lublin; PS, pharmacy students.
