# Supplementary material for: Do biobanks need pharmacists? Support of pharmacy students to biobanking of human biological material for pharmaceutical research and development
Source: Front Pharmacol. 2024 May 10;15:1406866. doi: 10.3389/fphar.2024.1406866 (PMC11117077; doi:10.3389/fphar.2024.1406866)
Supplement: Supplementary file 1 [file DataSheet1.docx]

Dear Students!

My name is Jan Domaradzki and I work at Poznan University of Medical Sciences. Together with my research team we are doing a project to better understand medical students’ knowledge and attitudes towards biobanking of human biological material for research purposes. We would like to invite you to share your opinion on this important topic.

This survey should only take up to 15 minutes of your time. At the same time, we assure that this survey is completely anonymous and confidential. As all responses to anonymous they cannot be traced back to the respondent. Moreover, while no personally identifiable information is captured your responses will be combined with those of many others and summarized in a report to further protect your anonymity. All information gathered will be used only for scientific purposes.

For any questions, or if you need assistance to complete this questionnaire, please contact:

Jan Domaradzki, dr hab. n. hum.

Department of Social Sciences and Humanities, Poznan University of Medical Sciences

Rokietnicka 7, Poznań, Poland

tel./fax: 61 8452 770; e-mail: [jandomar@ump.edu.pl](mailto:jandomar@ump.edu.pl)

Your feedback is very important. We appreciate your time and help

Sincerely

Jan Domaradzki

----------------------

**INFORED CONSENT FORM:**

1. I was informed by the Project Manager, Dr. hab. Jan Domaradzki about the planned scientific research, in particular about its assumptions, goals, course and method of conducting the research.

2. I understand all information provided to me regarding this research study.

3. I have been informed that participation in the research study is completely voluntary.

4. I have been informed that I can withdraw from participation in this research study at any time, without giving reasons, and my decision will not result in any penalties.

5. I have been informed that if I have any questions or doubts during the research study, I can contact the persons indicated in the information.

6. I voluntarily consent to participate in the study.

 Yes  No

----------------------

A biobank is a place where both hospital patients suffering from specific diseases and healthy people living in a given area can donate samples of biological material (e.g. body fluid or tissue) for research purposes in order to better understand health and diseases. These samples are also accompanied by other information and/or measurements (e.g. disease history, family history, lifestyle, height, body weight and composition, biochemical or hematological blood parameters, etc.), which is intended to allow for the best possible characterization of a given sample and understanding how lifestyle, environment and genes influence health

**1. Gender**

 Woman

 Man

**2. Year of study**

 1

 2

 3

 4

 5

**3. Place of study**

 the Poznan University of Medical Sciences

 Medical University of Lublin

**4. Place of residence**

 up to 10,000 inhabitants

 10-50,000 inhabitants

 51-100,000 inhabitants

 101-500,000 inhabitants

 above 500,000 inhabitants

**5. Significance of religion in personal life**

 very significant, it influences my decision and choices

 rather significant, I am guided by religious principles in everyday life

 small, I separate religion from public affairs

 it has no influence, it is indifferent to me 

**6. Have your ever heard about biobanks?**

 Yes

 No

**7. What are your impressions when you hear a word *biobank*?**

 Positive

 Negative

 Mixed, both positive and negative

 I do not know, it is irrelevant to me

**8. Should a research biobank collecting biological samples for research purposes be established in Poland?**

 definitely yes

 possibly yes

 possibly no

 definitely no

 I do not know

**9. If you were asked, would you donate your biological material to a biobank for research purposes?**

 definitely yes

 possibly yes

 possibly no

 definitely no

 I do not know

**10. What would be your primary motivation for donating your biological material to a biobank?**

 To benefit society and future generations

 To help progress science, help in generating new knowledge and development of therapies for various diseases

 To benefit my family, relatives and mine own

 The desire to receive medical treatment / service

 The desire to know my health status

 The desire to receive financial gratification

**11. What would you expect in exchange of donation samples of your biological material to a research biobank?**

 acknowledgments

 research results

 personal health information

 financial gratification

 nothing

**12. Do you think donors** **should receive financial compensation for donating samples?**

 Yes

 No

 I do not know

**13. Who should profit from the biobank research?**

 Sponsor of the research / biobank owner

 Donors

 Both biobank and donor

 I do not know

**14. Who should own the rights to the samples donated to the biobank?**

 Biobank

 Donors

 Both biobank and donor

 I do not know

**15. Would you donate your cancer tissues left over after a medical procedure to:**

 Research on the pathogenesis of cancer

 Research on curable somatic disease

 Research on psychiatric disorders, i.e. schizophrenia, depression

 Research on intelligence

 Research on aggression and violence

 Research on reproductive cloning

 Research into incurable genetic diseases

 Commercial research

**16. Would you donate the sample of your biological material to a research biobank led by**:

 medical university

 public clinical hospital

 private clinical hospital

 Polish biobank

 foreign biobank

 private biobank

 public biobank

 Polish pharmaceutical company

 foreign pharmaceutical company

**17. Would you donate to a research biobank if your biological samples would be accessible to:**

 only researchers from the institution one donated to

 researchers from Polish scientific institutions

 researchers from Polish commercial companies, including pharmaceutical

 researchers from foreign scientific institutions

 researchers from foreign commercial companies, including pharmaceutical industry

 Polish and foreign insurance companies
